# A return to lived experiencers themselves: Participatory action research of and by psychosocial clubhouse members

**DOI:** 10.3389/fpsyg.2022.962137

**Published:** 2023-01-04

**Authors:** Lester Vescey, Jennifer Yoon, Kevin Rice, Members of PARCO Group, Larry Davidson, Miraj Desai

**Affiliations:** ^1^Fountain House, New York, NY, United States; ^2^Program for Recovery and Community Health, Yale Department of Psychiatry, New Haven, CT, United States

**Keywords:** participatory research, clubhouse, mental illness, phenomenology, psychosocial rehabilitation

## Abstract

**Introduction:**

Within the history of psychology and phenomenology, people with lived experience of mental illness have often served as participants in research, but far less as co-researchers themselves. There is now a growing movement focused on “participatory” research, where people with lived experience directly contribute to various stages of the research process. This article presents such a qualitative, participatory research study, led by members of a large psychosocial rehabilitation clubhouse—Fountain House in New York City—and informed by phenomenological research principles. The study focused on collaboratively assessing and improving the clubhouse program for its members.

**Methods:**

A key feature of the project was the extent of lived experiencer involvement, for instance, in designing and conducting the study, and co-writing this research report. Members of Fountain House were trained in phenomenologically-informed research methods and developed a research study that focused on the quality improvement of their clubhouse program. Member researchers conducted a series of focus groups with fellow clubhouse members, generating qualitative data that were analyzed and written up by member researchers in collaboration with staff and university partners.

**Results:**

Overall, study findings place emphasis on the theme of action in members’ experiences—both with respect to how action, agency, and valued activity were key drivers of meaning and recovery for people facing severe mental illness, and with respect to the key component of the research process itself [i.e., participatory action research (PAR)]. Four major subthemes emerged from the study. First, findings revealed how members with mental illness experienced the clubhouse as a “new hope” and “the place for me,” to counteract their experience of inactivity, stigma, depression, and hopelessness prior to that point. Second, findings showed how, as members’ life goals changed, so did the precise meaning and role of Fountain House in their lives. Third, findings portrayed members’ need for, and pursuit of, transformation within the clubhouse space itself to provide more opportunities for meaningful work rather than what they viewed as merely busy-work. Finally, member researchers viewed their direct participation in this project as an opportunity to actively combat stigma, to be a driver of research, and to engage in what they viewed as a generative activity.

**Discussion:**

These action-oriented themes serve as a counter to the historical view of people with mental illness as merely passive experiencers of symptoms and passive recipients of mental health care. We discuss how the process and content of participatory research can help enhance the relevance of research for stakeholders’ lives and contexts.

## 1 Introduction

Persons with lived experience of mental health conditions have long advocated for research and knowledge about mental health to better reflect the realities of their own everyday lives. Indeed, people who have histories of mental illness are taking more active roles in studies pertaining to them, consistent with the rallying cry of the disability rights movement of “nothing about us, without us” (Charlton, 1998). One central way that greater inclusion and representation is taking hold is through the direct participation of persons with lived experience on study teams and research projects (Wallcraft et al., 2009; Davidson et al., 2010; Case et al., 2014; Desai et al., 2019). Participation in all aspects of the research by the community of interest as evaluators and collaborating investigators not only contributes to the empowerment of the persons within the community but also improves the validity and relevance of research findings (Hancock et al., 2012). A formalized research method that embodies these participatory principles is known as participatory action research (PAR), which acknowledges that expertise comes in many forms and seeks to involve members of the community of interest from the very beginning of developing research projects, all toward actionable ends (Lawson et al., 2015; Kidd et al., 2018; Chevalier and Buckles, 2019; Israel et al., 2019).

Settings within the mental health landscape that may be especially well-suited to PAR are mental health clubhouses (Pernice-Duca and Onaga, 2009; Hancock et al., 2012; Pardi and Willis, 2018). Clubhouses are intentional recovery communities informed by the rehabilitation principles of community integration, personal empowerment, and access to social support. These communities, which exist in over 300 locations around the world, invite people with histories of severe mental health challenges (e.g., schizophrenia spectrum disorders, affective disorders, and other severe psychiatric conditions) to work in unison with professional staff to carry out the duties, maintenance, and organization of the clubhouse milieu therapeutic model (Doyle et al., 2013). Thus, the underlying tenets of the clubhouse model share many principles with the PAR ethos (McKay et al., 2018), seeking to combat stigma in mental illness by empowering its members (not “patients” or “clients”) to participate in and take ownership of their recovery community by achieving the social, financial, and vocational goals of the membership through mutual and staff support (House, 1999).

Since 2017, members of the original mental health clubhouse, Fountain House in New York City, have been engaged in a PAR training and research project. The long-term goal of this project was to develop a member-led evaluation component within Fountain House that can illuminate the experiences of members and improve the quality of the clubhouse itself. The project has featured members being trained in and then conducting all aspects of a qualitative research study, informed by phenomenological research principles (Davidson, 2003). This includes designing interview questions, conducting and transcribing interviews, analyzing the narrative data, and leading group discussions to synthesize and create a report of the results. We further describe the relationship of phenomenology to our specific research approach in the section “2.3 Analysis.” A related quantitative survey study was also conducted and will be reported on elsewhere. This initial training and research project has led to the formation of a standalone Research and Knowledge Team at Fountain House that continues to pursue member-led participatory projects.

This paper represents a robust step in the process of clubhouse members conducting their own research and providing direction to the clubhouse for what will improve their lives and the lives of their fellow members. The approach may differ from many extant psychological and psychiatric studies, including phenomenological ones, given the sheer extent of the involvement of people with mental illness. In the present study, the member researchers themselves conducted focus groups with Fountain House members, focusing on topics selected by the participating member researchers, with a final topic selected by members of the Fountain House Board (as described in section 2.2). After conducting the member-led focus groups, Fountain House members trained in qualitative research methods analyzed transcripts of the focus groups. The participating member researchers then organized their findings into narrative summaries that elaborated the salient, consistent, and unique themes discussed in the focus groups. The findings of this initial study are summarized in the present paper, co-written by clubhouse members, staff, and university-based partners.

## 2 Materials and methods

### 2.1 Setting

The project was conducted at Fountain House in New York City and received ethical approval from both Fountain House and the university-based partners’ institution. Founded in the 1940s, Fountain House originated the clubhouse model of mental health, seeking to combat the stigma of mental illness by helping members achieve their social, financial, and vocational goals through an evidence-based model of empowerment, mutual support, and (co)operative administration of the clubhouse community (House, 1999; Gold et al., 2016; Raeburn et al., 2016; Hwang et al., 2017). Membership at Fountain House is voluntary, available for life, and is offered to any person with a history of serious mental illness. There are on average 1,300 active members of Fountain House, often with diagnoses of schizophrenia, schizoaffective, bipolar, and major depressive disorder.

Fountain House programs include internal research units, employment supports, administration, health and wellness, supported education, housing assistance, and other social supports idiosyncratic to each of the over 300 credentialed clubhouses in the United States and around the world (for credentialing criteria see Propst, 1992). Central to the clubhouse model is that members participate in the operations and decision making of the clubhouse program through a structured “work-ordered-day,” wherein they join a unit or department within the clubhouse (e.g., research, wellness, education, employment, etc.) to work side-by-side with peers and professional staff in conducting the unit’s services and operations (Tanaka and Davidson, 2015). These facilitated experiences of shared contribution drive what the clubhouse calls the *need to be needed* (Mancini, 2006), with a focus on rehabilitating member agency, self-confidence, and social acceptance through the structured development of social relationships and community stakeholdership. This therapeutic model of clubhouses is known as social practice, wherein peers and staff leverage the shared work, administration, and social relationships of the community to engage members in their own recovery while combatting the debilitating social stigma and isolation that many with severe mental illness endure in life (Sacks, 2009).

### 2.2 Procedure

Over the course of one academic year in 2019, members of the Fountain House research unit were trained in the methods of developing research questions, conducting focus groups, and applying qualitative analysis, by university-based researchers with extensive combined experience in qualitative, phenomenological, and participatory research. Participating member researchers first decided what issues they wanted to investigate and communicated these research questions to the university-based team of trainers. The university-based trainers supported participating members in the recruitment of voluntary participants for focus groups through announcements and sign-ups made at a community meeting held weekly at Fountain House. Inclusion criteria were that participants must be a member of Fountain House, be older than 18 years of age, and be capable of providing informed consent. A total of 12 members participated in the study ranging in age from 18 to 70 and ranging in length of membership from under a year to decades. They provided their informed consent to participate in the study. Each participant, by virtue of their being a clubhouse member, had a history of mental illness. Specific diagnoses for each participant were not recorded as the clubhouse model does not differentiate its services based upon a person’s specific diagnosis, and since it was fellow members leading the research study, disclosing this information was deemed as unnecessary and a privacy issue amongst fellow clubhouse community members.

Four separate 1-hour-long focus group sessions were conducted, each led by two interviewers who were members participating in the Fountain House PAR initiative. Participant group size in each session ranged from two to five interviewees. The focus group procedure was developed by the participating member interviewers, who were trained on interviewing techniques for qualitative research studies. The focus group procedure was structured as an open discussion surrounding the following core questions (the first two chosen by members and the final chosen by the Fountain House Board):

1. How has your life changed since coming to Fountain House?
  a. How might your life be different now if you had never joined Fountain House?
2. How do you relate to staff at Fountain House?
  a. How do staff relate to you?
  b. How do grievances get handled?
  c. How do you find working side-by-side here different than other places?
3. What do you hope to get out of Fountain House?
  a. Do you see Fountain House more as a destination or as a stepping stone?
  b. Where would you like to be in 2 years?

Interviews were recorded on secure devices and were later transcribed by members of the research unit who did not serve as interviewers. After removing all identifying information, the transcripts were then analyzed by Fountain House members in the PAR working group, who identified salient themes and narratives that emerged in the interviews through the lens of shared lived experience.

### 2.3 Analysis

Analysis was conducted *via* two phases. The first phase involved identification of themes within transcripts that stood out due to the salient meanings attributed by participants or to the level of occurrence. Consensus coding was conducted for each transcript where at least three member researchers, supervised by a professional research staff, would review the data independently and then convene as a group to review and select agreed upon themes. After consensus coding was completed, each transcript was reduced to an agreed upon one-page narrative summary that wove the salient, essential themes together into a more general story. In the second phase, these four summaries and their identified themes and illustrative data were transformed into a singular narrative summary that captured the core meanings of themes. This stage featured quoting, combining, and paraphrasing data from separate focus group transcripts into a single coherent structure (Sells et al., 2004; Malterud, 2012). The following four thematic areas comprise this overall summary, as they were identified by the analytic team as the most salient from the data, with a particular eye toward transformative action and quality improvement potentials.

We offer a final note pertaining to how this study was informed by phenomenological research principles. To begin, the two main trainers of member researchers were phenomenologists, with the associated technical assistance following from that general positionality. In concrete terms, this meant that the data typically emphasized concrete examples and experiences; analyses typically focused on the meanings of experiences in community contexts; and the findings sought a degree generality thorough delineating core features that encompass members’ experiences (Davidson, 2003; Wertz, 2005). Further, the study followed various established procedures for conducting phenomenological research, including the transmutation of lengthy raw transcripts into a one-page synthesis of essential major experienced meanings (Sells et al., 2004; Malterud, 2012). It should be noted, however, that the member researcher team developed their own innovative and hybrid approach to the above, infused by the member-driven and communal ethos of the clubhouse. As a prime example of this innovation, in the creation of one-page syntheses, the analysis group created a general story that incorporated *individual* focus group participant statements; however, when read as a whole, it provided a sense of a *community* experience, that is, of a community *experiencing*. In closing, we do not make claims to pure adherence to formalized phenomenological psychological methods, due in part to our extensive utilization of community-led methods. Yet, we do contend that the spirit of phenomenology—which was always supposed to be about the matters themselves or the “grassroots” (Spiegelberg, 1972, p. xlv)—has remained.

## 3 Results

Overall, study findings place emphasis on the theme of *action* in members’ experiences—with respect to how action, agency, and valued activity were key components of meaning and recovery for people facing severe mental illness, and with respect to the key component of the research process itself (i.e., PAR). These higher order themes of action, meaningful activity, and the active pursuit of transformation of their lived spaces were threads that ran through the more specific findings of the project regarding members’ temporal experiences of the clubhouse. By temporal experiences, we refer to their experience of life *before* the clubhouse, *during* the clubhouse, and *possible transformations of* the clubhouse, and clubhouse model, to better serve its members.

### 3.1 Driving research, dismantling stigmas, and creating new opportunities for marginalized groups

Member participants described various reasons for initially joining the PAR project, including the potential to be an active driver of research, to actively dismantle stigmas, and to create new opportunities for people facing marginalization and exclusion. Some members had a background in research, one of whom explained their participation by stating “I was very interested that mentally ill people could research mental illness ourselves. We are the focus of the research, so why not be the drivers of the research. I had a research background before my diagnosis, and so this served as an opportunity to get back into this world.” Other members described wanting to be a part of the group because of its contribution in combating stigma, with one person stating: “I did not have any background in research, but I was interested in the class because I could develop my skills to become a researcher to help in the work of dismantling stigmas and create new opportunities for marginalized groups.” However, some members’ reasons for joining emphasized a more basic sense of being able to participate in a meaningful, productive, and generative activity, with one member stating: “There was no expectation for myself in coming to the class. It merely served as an activity for re-engagement, to do something, because I was feeling that I wasn’t up to anything meaningful. All I knew is that I wanted to move on with something productive, to learn something.”

### 3.2 I would rather be at the clubhouse… than be in the hospital

The contrast between life before and after entering Fountain House was vividly conveyed by interview participants. For many, becoming a part of the clubhouse was a major milestone in participants’ lives. For them, it was a moment when the world opened up and “a new hope” came forth, filled with more meaningful activities and pursuits. Life before Fountain House often involved day-to-day struggles, a sense of purposelessness, depression, and isolation. As one person said, “[if I weren’t here] I’d just be sitting around.” The world outside the clubhouse presented all the challenges of unstable housing and prolonged unemployment, “running around on the streets,” and encounters with harassment, law enforcement, and repeated hospitalizations. Social rejection could be extreme, from strangers who would “think I was carrying a knife ‘cause I was mentally ill” to others who “might call the cops on [me].” Unlike these negative experiences in the outside world, participants described a welcome feeling of not having to explain themselves when coming to Fountain House as compared to everyday life previously. Respondents described relief from stigmatized interactions and relief at being accepted, even by themselves: “I’ve been able to accept what I am” and “I just feel very well liked and accepted here.”

Though the pitfalls of the world outside the clubhouse looked different to different people, a common sentiment about Fountain House emerged, relating to it being a place of possibility. This sentiment often contrasted with their experiences of hospital settings: “I’d rather be at the clubhouse… than be in the hospital… coming here is going to help me to do things I want to do with my life.” Members further described how their feeling of equal treatment and authenticity in member-staff relationships uniquely contributed to a feeling of egalitarianism and self-respect, “there are no doctors or therapists…members and staff are treated the same… like human beings.” Indeed, some members spoke directly of Fountain House being a preferred alternative to conventional mental health day programs, describing other programs as hierarchical, “they called ‘em clients there and they looked down on the clients. They considered them losers.” Alternatively, Fountain House was described as a preferred place to find respite and strive toward goals: “I haven’t been in the hospital in about 15 years,” said one participant, “the more Fountain Houses we have, the more hospitals will go out of business.”

In such terms, Fountain House was seen as a radical contrast to the stigma and social limitations they often faced as a person with severe mental illness: “It’s an amazing place, there’s no other like it in the world… we have people from different countries come all the time to find out our paradigm.” It was a place where members could meet friends to see each day and a path toward stability in areas like housing and health care. Said one participant: “Years ago I didn’t have many friends. I think I’ve come far now.” Relationships with staff were described as purpose-driven, egalitarian, accepting, and humane by many of the members: “Everybody’s equal here. No matter what the illness, what diagnosis, they treat you like a human being… treat me as important… I really feel as if I have something to offer.” The person’s world expanded from just oneself to a network of treasured relationships with peers and staff, working side-by-side each day, leading to a profound sense of belonging: “This is the place for me.”

### 3.3 Needing more than just a place to go

Within the overall expression of belonging amongst members of Fountain House, there was also a sentiment about needing more than just a place to go, that meaningful opportunities were required for deeper fulfillment, beyond just being there. Some felt Fountain House fulfilled this desire, whereas others felt it came up short, particularly with regard to being able to provide opportunities for creative expression or impactful work. Initially positive experiences in the community could eventually wear off, with one member stating:

*Fountain House gives me camaraderie and respite from some problems. However, I feel like I am not intellectually stimulated anymore. When I started at Fountain House everything was new and stimulating, and if I stayed home I would crash. For me it got me out of the house, but now I’m bored during the work-ordered day. It is a bit difficult for me to engage, and I feel like my creative self is not allowed to be expressed. Instead, I feel like I am being pressured to do busy work, I feel thwarted.*

Other members went as far as to describe some experiences in the work-ordered day as a “sheltered workshop.” However, these same members went on to describe a scenario in which they were able to become engaged in an important task that arose when trees were knocked down in the backyard. Helping out on this task provided these same members with an opportunity to work collectively on a meaningful task to the benefit of the whole clubhouse, indicating that while certain work did not appeal to them, the clubhouse had other engaging opportunities for contribution. “There was a more worldly aspect to the task,” one member elaborated, “unlike the tasks of making bookmarks and stuff, which can get monotonous.”

Some members commented on experiences where they felt a need and desire for Fountain House to be more proactive with regards to overcoming and intervening in the persistent struggles many members face. As one member stated:

*I have a tendency to be someplace and somehow get stuck there, like I’m in quicksand and I can’t move out, so I wish Fountain House (FH) was more, that I was getting more assistance… I get ideas in my head and I can’t put them into action, and sometimes I feel like the staff can’t help me with that. I need help developing myself, more than just a place to come.*

Other members also spoke to this feeling, emphasizing that while they may not have as severe of needs as other members, their issues with mental health problems like depression still persisted and could go overlooked:

*I’m not homeless, I’m not suicidal, but I still struggle with my depression… I feel like those members who have more needs get prioritized, while those of us who have had some success in the past are told that those resources are not for us… There’s still room, especially for high functioning people, for improvement. I think we all want to be here for different reasons, but FH doesn’t work perfectly. Depression can make it very difficult to follow through, so I’m just saying it’s not just me personally but also my symptoms. The staff are also just not as available as I would like or the community needs.*

Many members spoke to the experience of staff unavailability, explicitly with regards to staff being taken out of the house by impromptu supported employment responsibilities known as transitional employment (TE), where staff must cover a position for an absent member, often rendering the former suddenly unavailable. As one member commented, “This TE thing that they have to respond to like that. She’s on a TE, he’s on a TE, she’s on a TE, and it’s just like by the time you get (to see the staff), you’re frustrated.” Other members spoke to this general frustration regarding staff having precarious schedules: “One is at the farm, one is in Alaska, and one is at diversity training. I could go up to 3 days without seeing my worker.” This frustration with the precarious availability of staff and workers was also reported as an evolving circumstance that has changed over the long history of Fountain House:

*When I first joined here, there wasn’t as much to do but I felt there was more of an intimacy between staff and members. I feel like maybe there could be more one-on-one time. I feel like maybe Fountain House could try to take some of the good qualities of what it used to be like. I would meet staff out for lunch together or do different things and I don’t feel that happens that much anymore, but I do feel that staff really care about their work, and they try to support me but they don’t really do therapy. There was more one-on-one time back then. The staff and I built a rapport together. They knew how I was doing in all aspects of my life.*

### 3.4 I figured out how to navigate Fountain House, what it can give, and what it can not

Members’ reports indicated the possibility of a dynamic recovery journey in their engagement with Fountain House, in that over time they navigated how to actively explore the supports within, manage the limitations of, and creatively contribute to the Fountain House community. As one participant described:

*I’ve had success this time around because I’ve figured out how to navigate Fountain House, what Fountain House can give to me and what it can’t, and where I need to go to seek out some other things and other sources of support. And again how I can better engage myself in this community so that I’m maximizing what I can bring to the community.*

Many participants echoed this agentic sentiment, speaking to their quest to be “proactive in (their) own health.” Members spoke to this experience of the clubhouse as a community where one is accepted upon showing up, even though not quite knowing one’s way, while subsequently engaged and invited toward finding a niche in the community.

Across other reports, it was also suggested that members had an evolving perspective on the role they saw Fountain House foreseeably playing in their life: as their own life goals and trajectory shifted, so did the nature of their relationship with the clubhouse community and setting. As one member stated regarding not needing the supports of Fountain House as much as they used to: “I wouldn’t want to leave FH but I may engage with it differently.” Another member elaborated on this point, stating:

*It gives me structure even though I don’t need Fountain House as much I did before… In this way, Fountain House is both a stepping stone and a destination for me… I feel like I’ve made some very strong relationships with members as well as staff working on those projects and it makes me feel like I can accomplish things…[but] I still need their ability to keep me congruent and on schedule… This is a place that I always want to volunteer and be able to come to, whether it’s a Thursday night or a work-ordered day, I see it as a part of my life…for a long time.*

It appears that the development of supportive relationships and a reliable space of welcoming were key to what motivates and keeps people engaged with Fountain House: “I have a lot of friends who I met at Fountain House. Years ago, I didn’t have many friends. I think I’ve come far now… I want to be more social. I want to achieve.” One particular exchange between two members effectively highlights the experience of one’s relationship with Fountain House evolving over time:

*P.1: For me, for a long time, I felt like I wasn’t getting anything out of it. I would leave for sure and [not] come back for years. In this go around, I feel like I made some very strong relationships. With peers as well as staff, and it’s staff that has helped me to stay. In those relationships and talking to people in the last year I kind of figured out where I could be effective here in this community while using my skills in this community and I started to do that.*

*P.2: But what about outside of Fountain House–I assume you want to stay. I mean you will always be a member of Fountain House but from what I heard from you, you don’t want to be here in 20 years. So what about leaning in that direction with what you have you gotten from Fountain House?*

*P.1: So it’s a matter of the relationships I made here… [The staff are] so encouraging and supportive, and that has been helpful. To me in 20 years, do I want my work with Fountain House to look differently? Of course. I don’t know if I want to leave the Fountain House community in 20 years. I think there’s something valuable here for me in terms of breaking isolation. In terms of coming to a community where I don’t have to explain things and that’s important to me.*

Others described the persistence of Fountain House in their lives through various life events: “I haven’t been in the hospital for 8 or 9 years I think, and I had a pretty good streak here the last 6 years… I graduated college… I worked… I feel like my mental health has improved a lot.”

Though members’ relationship to Fountain House continually evolved, they also described how it can become a lifelong fixture in their lives—changing in meaning and need but stable in presence. “I’d like to stay connected and I’ve been here for so long it’s almost like a part of my life now and I feel like I’m kind of a lifetime member because I’ve been here for so long [since the late ‘80s right after high school] and I’ve come back. I’ve went away and left Fountain House and did my own thing and then I valued Fountain House enough to come back…” One member reported having integrated Fountain House into their life, and described it as, “a place that I always want to volunteer,” as well as being a place they would always want to engage with. “I wouldn’t leave the Fountain House community but I may engage with it differently whether or not I’m engaged with the community or working full time successfully.” Another member was not sure where they would be in 2 years, but they could say that Fountain House would still be a part of their lives. It is a place that they always want to come to, “whether it’s just a creative writing group Thursday night, or just being part of the work-ordered day… I see it as a place that is part of my life, and that is an important part of it.”

## 4 Discussion

This PAR study, featuring the direct contribution of persons with mental illness in all aspects of the research process, sought to better understand and improve members’ experience of everyday life in a prominent psychosocial clubhouse. The ensuing study findings emphasize the centrality of *action*, *agency*, and *meaningful activity* in the lives and recovery journeys of people with mental illness. In the focus groups, members reported experiencing the clubhouse as a space of possibility, hope, and belonging, which contrasted with their experience of inactivity, stigma, depression, and hopelessness prior to that point (Section 3.2 “I would rather be at the clubhouse… than be in the hospital”). Findings also portrayed members’ need for, and pursuit of, *transformation* within the clubhouse space itself to provide more opportunities for meaningful work and staff engagement, rather than what they sometimes viewed, for instance, as merely busy-work (Section “3.3 Needing more than just a place to go”). In addition, findings showed how such a hospitable, non-hospital setting did not hold a uniform meaning across time for members with mental illness: meanings shifted relative to the specificities of their own *agentic* goals and life-historical trajectory (e.g., whether it was experienced as a stepping stone or a final destination) (Section “3.4 I figured out how to navigate Fountain House what it can give, and what it can’ not”). Finally, although it was not formally studied in the current project, a general consensus of members who contributed as researchers in the study (some of whom are authors of the current manuscript) noted how direct participation in this project *as a PARer* afforded opportunities to actively combat stigma, to be a driver of research, and to engage in what they viewed as a generative activity (Section “3.1 Driving research, dismantling stigmas, and creating new opportunities for marginalized groups”).

The portrait of action and activity illustrated above contrasts with those found in the history of psychology and psychiatry that have—either implicitly or explicitly—often placed more emphasis on passivity, for instance, that people living with mental health disorders are largely passive experiencers of symptoms and passive recipients of treatment. Variations of these themes have also been subtly repeated within the strands of phenomenological psychopathology that, in their descriptions of the (mostly internal) world of mental illness, have tended to lose sight of the person and their surrounding context, or, further, to describe these as largely passively experienced illnesses or as deviations from a more normative being-in-the-world (Davidson et al., 2004). However, such a phenomenological psychology, as Husserl (1954/1970, 1981) suggested, may unwittingly take for granted what appears as “person,” “illness,” and “world” rather than inquiring into their very constitution, including such surrounding notions as “normal” and “abnormal,” “healthy” and “pathological.” Further, such a world, rather than being defined merely by passive reception, is always actively being constituted on intersubjective grounds, albeit with the challenge posed by previously sedimented meanings. What this means is that the lived experiences of people with mental illness cannot be separated from the constitution of the world, inclusive of the norms, processes, prejudices, and possibilities therein, and of the active contestations of each (Davidson and Cosgrove, 2002; Davidson, 2003; Jenkins and Carpenter-Song, 2009). What this also means is that the current state of affairs may not be the only state of affairs as activity and action are possible, both individual and collective, including the activity of recovery, and the roles of persons, structures, and institutions within it. Discussion of such intersubjective processes being facilitated in clubhouse program settings has been explored in relation to positively impacting recovery trajectories in serious mental illness (Rice et al., 2020). One implication for the broader study of *mental* health and illness is to more closely interrogate the *social*, *institutional*, and *community* structures around the person, as potential contributors to illness and to recovery. Otherwise, the risk remains of locating the determinants of mental health, agency, and activity as solely inside the person, with psychology in danger of becoming a world unto itself, divorced from the real world, out there; the latter bias would be a variation of psychologism, the overcoming of which has always been a central aim of phenomenology (Desai, 2018; Davidson, 2021).

### 4.1 Practice and policy implications

This PAR project, staffed and infused with member energy and participation, examined the experience of Fountain House from members’ perspectives. The goal was to produce actionable insights that could lead to community improvement. Part of the challenge of elucidating targets for intervention was that Fountain House was not experienced as a singular thing. It appeared differently to different members, and for any individual member, its function and role in their lives could change over time. The meaning of Fountain House as a place and communal space evolved with the lives of longtime and new members creating a constellation of needs and services that were dynamic to the constitution of the membership, regardless of whether the building and staff stayed the same. This was the first main implication of our findings, that the meaning and role of “Fountain House” may evolve over time for members, their relationships, interactions, and modes of giving and receiving. In some cases, it was even less of a place as such, but a stable presence in one’s life that members carry with them and know they can return to in times of need.

Within the expansive range of what Fountain House could be in a person’s life, members could come to experience the clubhouse as becoming a true home that meets their needs and desired roles, or, as one participant described, simply being the “place for me.” Many members described their initial draw to Fountain House as being due to the clubhouse sense of community and engagement disrupting previously persistent experiences of loneliness and disenfranchisement, a finding consistent with other recent studies on the impact of community and the “oasis”-like qualities of the clubhouse model (e.g., Conrad-Garrisi and Pernice-Duca, 2013; Mandiberg and Edwards, 2013; Kinn et al., 2018; Tanaka et al., 2018; Desai et al., 2021). However, this was not the automatic or continual case for everyone. This was highlighted in the examples of some members experiencing frustration in having their needs met. Such experiences draw attention to possible revisions Fountain House may administratively and communally undergo for the sake of either meeting the evolving needs, or setting clearer expectations, of its dynamic membership. This focus on unmet needs coincides with our action-oriented framework intended toward identifying avenues of potentially beneficial change. We believe that one of the major sources of contribution of this project to the wider literature on clubhouses and psychosocial research is in being able to identify areas of improvement, in a respectful and yet constructive manner—insights that were greatly facilitated by the project being member-driven and member-to-member run. We break these aspects down into three main domains: boredom/stuckness; nature of work; and staff engagement.

The above evidence suggests that members can come to find that, while the initial period of engagement may be characterized by expansion, opening, and excitement, this momentum can slow down, bringing members to experience a sense of stuckness, boredom, or stifled creativity. This experience is directly tied to the nature and quality of the everyday work experience. Members found that there were important differences in the meaning and enjoyment of work, depending on the type of work, the background of the member, and the synergy between the two. Experiences varied in terms of how extensively a member perceived it as, for instance, drawing on their creative side or as impacting real concerns in the world. One member offered the quite provocative, critical description of coming to view the work-ordered day as creating a “sheltered workshop,” which illustrates the range of members’ varying views of this core clubhouse feature and its possible discontents. Greater attention by clubhouses to facilitating more meaningful and generative activities for these members—with direct member input—may be warranted. During the discussion of these findings with the study team, there was also consideration of the cultural implications of work, that is, whether the kinds of work available in the broader economic structure of the US, in general, may often contrast with the important clubhouse goals of developing relationship, community, and purpose.

Members also differ with respect to their experience of staff engagement. Members can come to want more engagement opportunities with staff, particularly when staff are often called away to other obligations. The clubhouse model requires a deliberate understaffing policy so that the clubhouse could not be maintained without the contributions of its members (Propst, 1992), but there may be cause for some reconsideration of this in light of members’ feedback. However, previous research on member-staff relationship done by Pernice-Duca (2010) found that relationships with other members lead to greater improvements in mental health than receiving support from staff, for which the deliberate understaffing model of Fountain House may encourage member-to-member support. At the very least, the policy of deliberate understaffing could be better explained to members, which would improve transparency and potentially limit disappointment, or be supplemented by formally designating experienced peer-members as support staff. Such findings also bear comparison to previous research on the difference between clubhouse staff being more likely to describe the clubhouse community in terms of broad standards and values (Herman et al., 2005), whereas member perceptions focus more on staff-member relationships, program empowerment, and the importance of work (Burt et al., 1998).

There were several limitations of this research. First, while we attempted to produce qualitative themes about experiences of Fountain House, we do not claim that these themes capture all of the unmet needs that members may face. However, we do suggest that these themes are pertinent, given the contributions of the member-stakeholders themselves to the study. Additional research is required to further explore these and new, arising areas of concern, including specifying the full determinants of robust action and agency among community members. Second, as this was a qualitative study describing experiential themes, future research would need to determine the quantitative frequency of specific experiences described in this study, as well as the relation of specific themes to factors like membership length and other key demographics. Third, there may have been a selection bias toward members who were verbal and active, and against those who may not be as engaged in the clubhouse in general. Fourth, Fountain House is a large clubhouse program with a variety of support services and contexts; our findings and quality improvement suggestions should thus be viewed within the context of relatively small proportion of participants in relation to a large total membership and within the context of a large program like Fountain House (i.e., they may not be representative of member experiences in smaller clubhouse programs).

## 5 Conclusion

This paper demonstrates the unique contributions that can be developed by members in therapeutic communities conducting research on themselves for themselves. Such research not only helps provide insight into the experiences of mental health service beneficiaries, but also provides a concrete mechanism for those experiences to be represented in research methods, qualitative analysis, and program evaluation. The research being conducted by the community, for the community, directly benefits life at Fountain House by providing constructive feedback from the main beneficiaries of the clubhouse model, the members. It also provides an opportunity for member researchers to develop a research skillset that can be applied in the pursuit of future community interests and action.

## Data availability statement

The datasets presented in this article are not readily available because they are qualitative in nature, and there is a need to preserve anonymity and confidentiality among participants and the organization. However, key data are presented directly in the text. Requests to access the datasets should be directed to KR, krice@fountainhouse.org.

## Ethics statement

The studies involving human participants were reviewed and approved by the Fountain House IRB. The patients/participants provided their written informed consent to participate in this study.

## Author contributions

Members of PARCO Group were members of the Fountain House Research Unit and community who contributed to the design, implementation, and analysis of the research project. All authors listed have made a substantial, direct, and intellectual contribution to the work, and approved it for publication.
